# Pushing poverty off limits: quality improvement and the architecture of healthcare values

**DOI:** 10.1186/s12910-021-00655-x

**Published:** 2021-07-13

**Authors:** Polly Mitchell, Alan Cribb, Vikki Entwistle, Guddi Singh

**Affiliations:** 1 Centre for Public Policy Research, School of Education, Communication and Society, King’s College London, Waterloo Bridge Wing, Franklin-Wilkins Building, Waterloo Road, London, SE1 9NH UK; 2 Health Services Research Unit and School of Divinity, History and Philosophy, University of Aberdeen, 3rd floor, Health Sciences Building, Foresterhill, Aberdeen, AB25 2ZD UK; 3 Mary Sheridan Centre for Child Health, Guy’s and St. Thomas’ NHS Foundation Trust, 5 Dugard Way, London, SE11 4TH UK

**Keywords:** Healthcare values, Healthcare quality, QI, Quality improvement, Social determinants of health, Poverty, Screening

## Abstract

**Background:**

Poverty and social deprivation have adverse effects on health outcomes and place a significant burden on healthcare systems. There are some actions that can be taken to tackle them from within healthcare institutions, but clinicians who seek to make frontline services more responsive to the social determinants of health and the social context of people’s lives can face a range of ethical challenges. We summarise and consider a case in which clinicians introduced a poverty screening initiative (PSI) into paediatric practice using the discourse and methodology of healthcare quality improvement (QI).

**Discussion:**

Whilst suggesting that interventions like the PSI are a potentially valuable extension of clinical roles, which take advantage of the unique affordances of clinical settings, we argue that there is a tendency for such settings to continuously reproduce a narrower set of norms. We illustrate how the framing of an initiative as QI can help legitimate and secure funding for practical efforts to help address social ends from within clinical service, but also how it can constrain and disguise the value of this work. A combination of methodological emphases within QI and managerialism within healthcare institutions leads to the prioritisation, often implicitly, of a limited set of aims and governing values for healthcare. This can act as an obstacle to a genuine broadening of the clinical agenda, reinforcing norms of clinical practice that effectively push poverty ‘off limits.’ We set out the ethical dilemmas facing clinicians who seek to navigate this landscape in order to address poverty and the social determinants of health.

**Conclusions:**

We suggest that reclaiming QI as a more deliberative tool that is sensitive to these ethical dilemmas can enable managers, clinicians and patients to pursue health-related values and ends, broadly conceived, as part of an expansive range of social and personal goods.

## Background

Both primary and secondary healthcare predominantly treat and manage the downstream effects of social deprivation, rather than tackling its upstream causes. This limits their ability to alleviate either social deprivation or its effects on population health and health services in the long term. Nonetheless, many healthcare professionals recognise the immense health and social burden of poverty and social deprivation, and some are keen to harness the resources available to them in order to take effective action to tackle social deprivation and its contributions to health problems more directly from within healthcare institutions. This paper explores some of the ethical challenges facing clinical professionals who seek to expand their clinical roles to address the social determinants of health.^1^

Our discussion draws on a case in which clinicians introduced a poverty screening initiative into paediatric practice using the discourse and methodology of health service quality improvement (QI). Clinicians paying attention to and doing something to help address social deprivation could be seen as a way of extending conceptions of good quality care and strengthening service quality. But we argue that while using a QI frame can help legitimise interventions and secure funding, it can also constrain and disguise their value. We explore and illustrate how a combination of methodological emphases within QI and managerialism within healthcare institutions leads to the prioritisation, often implicitly, of a limited set of aims and governing values for healthcare. These norms and values structure expectations about what it is that clinicians should be doing, and can act as an obstacle to a genuine broadening of the clinical agenda. They tend to reinforce norms of clinical practice which closely prescribe ‘health-related’ outcomes and effectively push poverty ‘off limits.’ We set out the ethical dilemmas facing clinicians who seek to navigate this landscape in order to address poverty and the social determinants of health. We suggest that reclaiming QI as a more deliberative tool that is sensitive to these ethical dilemmas can enable managers, clinicians and patients to pursue health-related values and ends, broadly conceived, as part of an expansive range of social and personal goods.

Our discussion builds on, and to some extent maps onto, the well-trodden territory of value pluralism in healthcare, and in particular the tensions between different values and goals in this domain. But our focus in this paper is not on resolving these tensions, or suggesting conditions for their resolution. Rather, we are interested in illustrating and analysing the normative complexity that is generated when these tensions are negotiated in practice. Specifically, we consider the way that different values are reflected in and endorsed by the practices and actions of differently placed people—clinicians, patients, managers. We suggest that there is a tendency for a limited set of values to be continuously reproduced in clinical settings because of the literal and conceptual inaccessibility of certain objects of value to powerful actors.

### Case study: poverty screening initiative

Our case study is a poverty screening initiative—henceforth PSI—developed in a paediatric assessment unit in a London (England) district general hospital. The PSI seeks to empower clinicians to be more attentive and responsive to the social contexts of patients and their families, and to help them access resources and services locally. As a bottom-up initiative, spearheaded by clinicians, the PSI makes use of the resources and tools that clinicians have available to them, rather than attempting to enact high-level institutional change. The intervention comprises a short clinical screening survey and an informal referral process [4], reflecting recognition that ‘screening and referral’ is widely invoked and well-evidenced as an approach for front-line clinicians to contribute to the mitigation of social deprivation and poverty [5–12].

The PSI survey is completed by a patient’s parent or guardian while waiting to be seen, and given to their attending clinician. It includes questions about key markers of poverty and social deprivation, such as transport issues, housing insecurity, unhealthy and unsafe home environments, challenges procuring food and essentials, and lack of emotional support. If any of the survey answers suggest cause for concern, clinicians are encouraged to initiate a conversation with the parent—during the consultation—about the patient and family’s personal and social circumstances. If appropriate, clinicians can provide parents with a leaflet and advice about accessing local resources and services aimed at increasing income and enabling access to essential goods and supportive local community initiatives. These include debt and benefit advice services, grant-awarding organisations, food banks, voucher schemes for essential items, housing charities, domestic violence services, playgroups and support groups. The survey answers enable clinicians to tailor the conversation and advice to family circumstances. Patients and their families are not formally referred to local services or enrolled in relevant programmes—rather these resources are signposted to parents, who can decide whether to initiate contact.

The PSI was developed by frontline clinical staff as a QI project. The clinicians who spearheaded the initiative deliberately positioned the PSI as a QI project, followed a QI methodology and evaluated its success using predefined measures in order to justify the project to their managers and colleagues. Without this QI framing they consider it very unlikely that the project would have secured institutional managerial permission to proceed. They also provided managers with evidence of the link between poverty and healthcare service use and the clinical benefits of screening and referral programmes, in order to justify the value of the project [10, 13–15].

QI projects typically seek to improve the quality of healthcare by measuring and evaluating current practice, and systematically introducing and evaluating interventions designed to improve upon it. The PSI screening tool and referral process were devised and refined via a series of Plan-Do-Study-Act (PDSA) cycles—a structured approach to trialling and fine-tuning a change in practice in order to assess its impact [16]. Initially, potential questions and conversation-starters for the clinical encounter were proposed and discussed by staff in the paediatric assessment unit. Clinical staff were introduced by their peers to the initiative via multi-disciplinary teaching sessions and a ‘how to ask about poverty’ guide was developed and circulated to all staff in the unit. A survey and resource leaflet were drafted, and subsequently tested and refined to ensure that they were appropriate, inoffensive, non-stigmatising and acceptable to both patients and clinicians. Consistent with the QI approach, the success of the PSI was formally assessed—in this case via a series of process and outcome measures: percentage of doctors who screened for child poverty; percentage of doctors aware of local resources for children and families living in poverty; number of poverty resource leaflets given per shift; and patient feedback [4]. Longer-term outcome measures were not captured.

The PSI intervention has gained momentum and spread quickly to other sites in London, including community services and other acute services. On the whole it has been spread by word of mouth and championed locally by clinicians, indicating that it feels both practicable and worthwhile to staff on the ground. Qualitative feedback from staff and patients reinforces this reading [17]. We are not seeking to further review or evaluate the success of the PSI here. Rather we are using it as a case study to analyse the affordances and constraints of clinical settings for attending to patients’ social contexts and experiences. In particular, we are interested in the ways that the architecture and language of healthcare quality and QI enable and constrain the promotion of different kinds of ends and values, and the choices and compromises clinicians have to make in order to navigate this domain.

We intend our arguments to have relevance outside of the English context, but our analysis of the case inevitably reflects the values and operation of the system within which the PSI sits. QI has a high profile and is well embedded in the English NHS. The long-term plan published in 2019 by NHS England and NHS Improvement—the body that leads the English NHS—has the improvement of care quality and outcomes over the following 10 years as one of its core goals [18]. NHS England and NHS Improvement also has a track record of building workforce capability and offering advice and training in QI methodologies. National quality initiatives include ‘Quality Accounts’—an indicator-focussed report that every provider of NHS healthcare services in England must publish annually—and ‘best practice tariffs, a quality-based payment system, with differential tariffs for hospital admissions depending on the quality of care provided [19, 20]. QI activity on the ground is uneven but commonplace and, although a range of QI approaches are practised, the Institute for Healthcare Improvement’s Model for Improvement—which uses the kind of PDSA cycles adopted in the PSI—has been particularly influential [21]. The conceptualisation of healthcare quality at the level of hospital management in England emphasises external factors, such as quantifiable indicators and national targets, and focuses on clinical measures of quality, suggesting a ‘managerial approach’ [22]. In recent years, attempts have been made to integrate health and social care—in 2018 the Department of Health was rebranded the Department of Health and Social Care—but over the past decades, attempts to tackle health inequalities via ‘joined-up government’ have struggled to overcome siloed units, and divergent priorities and targets [23].

The case study presented here takes the form of an ethical argument rather than a formal data-generating piece of empirical bioethics. However, in constructing our argument we sought to achieve some of the spirit and advantages of empirically informed work, and it is worth briefly elucidating the approach we took. One of the authors (GS) was the prime mover behind the PSI and simultaneously worked with the other authors to analyse some of the ethical issues arising from this QI initiative. This collaboration provided an extra dimension of professional and theoretical reflexivity on the initiative, in addition to the qualitative evaluation built into it. The process involved a series of four roundtable discussions in which the co-authors considered the genesis, enactment, experiences and challenges of the PSI. These discussions were informed by the first-hand reflections of the principal actor and reported accounts from other participants and stakeholders, as well as by the PSI documentation and evaluation paperwork. Between roundtable meetings authors shared notes and sketches of possible arguments for consideration, and began to identify analogous issues and themes in the broader literature. This process allowed for considerable back and forth between engagement with the concrete details of the initiative and the developing theoretical and literature-based analysis. It also generated the key themes reviewed in what follows—notably the tensions that arise both at the interface of the clinical and public health functions of healthcare services and between more managerial and deliberative models of QI.

## Discussion

### Addressing poverty within clinical practice

Before turning to these questions about the normative ‘architecture’ of healthcare quality, we think it is important to respond to potentially sceptical voices arguing that addressing poverty should simply fall outside the domain of clinical roles and ethics. We will stop short of arguing that there is a professional obligation for clinicians to address poverty, but we would argue that addressing social deprivation is of clear ethical relevance to clinicians and that there is also a *prima facie* legitimacy in clinicians exploring the specific contributions they can make in this area because of the distinctive affordances of clinical settings.

Poverty is obviously a challenge for those who experience it, powerfully undermining their personal and social lives. But it is also a considerable challenge for health services. Increased social deprivation is associated with higher rates of primary care consultation, higher numbers of medical referrals, and more emergency and psychiatric hospital admissions [14, 24]. The most deprived people are far more likely to be admitted to hospital over their lifetimes compared to the most affluent, with significantly greater associated lifetime costs despite lower life expectancy [13]. Formal health promotion and preventative services have some potential to moderate the adverse health implications of poverty on individuals and health services. However, uptake and impact rates of services such as cardiovascular checks, childhood immunisation and screening programmes are often lower in socially deprived groups, and the most deprived people may be less able to follow standard medical advice about healthy lifestyles [24, 25]. This can mean that potential health benefits are not realised, and social inequalities in health can even be exacerbated.

It is thus understandable that those frontline clinicians who are conscious about the social determinants of health can be motivated to use their knowledge and positioning to make a contribution to addressing the everyday challenges that their patients face. But it might be argued that addressing the social determinants of health is simply not a clinical responsibility. Some conservative voices, for example, might argue that clinicians—especially hospital-based clinicians such as those who developed the PSI—should concentrate their energies on those biomedical concerns for which they have specialised expertise and dedicated resources.^2^ Alternatively it might be argued by more progressive voices that engagement in such agendas is important and something doctors should embrace as citizens but in ways that effectively fall outside the clinical sphere, for example, through the exercise of political voice and lobbying in partnership with a range of other agencies and movements.

However, it is worth highlighting that frontline clinicians and clinical settings are in some ways peculiarly well suited to identifying markers of poverty and offering advice to patients [26]. Clinical relationships themselves have affordances that are relatively rare. There are established conventions and expectations that make it possible for clinicians to ask questions about a patient’s personal and social history and to offer help and advice, albeit within limits and recognising potential ethical concerns about invasiveness. An intervention such as PSI takes advantage of this fact. Its format and application as a familiar universal and ‘neutral’ screening tool helps to make it acceptable to clinicians and patients. Patients are familiar with being asked to fill in forms in the waiting room prior to their consultation, to be handed over to their attending clinician. Clinicians are familiar with quickly evaluating such forms and using them to direct and shape the clinical encounter. While there is something quite dispassionate and impersonal about filling in a form, the survey creates opportunities for deeply personal and potentially emotionally charged conversations. The tool enables the patient and their healthcare professional to share information in a way which ‘breaks the ice’ with respect to topics and issues that it might otherwise be socially awkward to raise. The tool itself, then, takes some of the impassive conventions of the collection of information within the context of clinical consultations, and uses this as a prompt for conversations which go beyond the normal content and scope of healthcare practice. It provides, then, a ‘peg’ on which to hang topics for discussion which are typically excluded from this space and, by so doing, allows a different set of values to be embodied in and promoted by the healthcare encounter—reflecting, for instance, a wider conception of health and different goals of healthcare.

The issues raised by the PSI and the questions and conversations to which it is intended to give rise may touch on topics which are rarely publicly broached—even with close friends and family members—such as income, financial security, and domestic violence. However, clinical spaces already carry the expectation that personal information will be discussed openly and confidentially. Discussions, topics and actions which might be ethically or emotionally risky in other contexts—asking someone to undress, touching their body, asking them about their sexual activity, alcohol intake, and diet—are in some respects made possible and safe within the context of clinical encounters. The anomalous conventions of the clinical encounter mean that, perhaps paradoxically, conversations about poverty and social deprivation in clinical contexts may be experienced as less threatening or stigmatising compared to analogous conversations being initiated in non-clinical contexts.

Of course, by the same token, the clinical setting and the design of the tool may render the resulting exchanges and relationships less flexible than is desirable. A patient’s expectation that clinical consultations will be chiefly concerned with their biomedical health might prevent them from feeling able to talk as freely about their domestic and financial circumstances with a clinician as they would with a social worker, for instance. However, for those people who live in poverty but are not deemed vulnerable enough to warrant their having a social worker—potentially a large number in times characterised by recession, economic stagnation and funding cuts to core social services—clinical encounters may be one of their only opportunities to discuss these topics with an informed professional. Interventions like the PSI may create objects of value for clinicians and patients in constrained circumstances, without these being ideal or optimally valuable. The use of a survey with a predetermined set of questions might constrain what both patients and clinicians deem relevant to or part of the social determinants of health. But the clinicians who developed the initiative saw the survey as a starting point for a conversation rather than a technical or exhaustive assessment of the social determinants of health. For these reasons interventions such as the PSI can plausibly be presented as ethically defensible utilisations and extensions of the clinical role, with the potential to support other public health initiatives which aim to relieve poverty.

### Valuing healthcare interventions

For the clinicians involved, the framing of the PSI as a quality improvement initiative was central to efforts to secure its perceived legitimacy. ‘Quality’ is a relatively open-ended and contested concept which, even on standard accounts, accommodates multiple dimensions (including equity and patient-centredness) [27, 28]. This makes a QI framing elastic enough to support a diversity of goods, values and aims in healthcare. However, in practice, the costs and benefits of interventions are not only very diverse in nature, but also differently understood and prioritised by differently placed actors. In this section, while acknowledging how using a QI frame can enable the pursuit of better healthcare, we draw out some of the complexities inherent in appeals to quality. We highlight the challenges of valuing the costs and benefits of healthcare interventions including the perspectival character of these valuations—the fact that different people value different goods and ends differently.

#### The costs and benefits of healthcare intervention

Interventions like the PSI have a range of foreseeable costs and benefits for different actors. If successful, patients experiencing the challenges of poverty will receive information about local resources and be able to access and use them. This will potentially lead to benefits such as increased income and household resources, safer and more healthy home environments, better emotional support, higher well-being and lower stress levels. These might lead to better health and healthcare outcomes for patients and their families, which, in turn, could lead to decreased or more effective use of healthcare services—reducing the burden on either the paediatric assessment unit or other parts of the system. This causal pathway is by no means a straightforward one, but research into the social determinants of health suggests it is at least possible [29]. There may also be more proximal benefits associated with the intervention—open and honest discussions between parents and clinicians about poverty might help to build trust and good communication—which can also plausibly contribute to the safety and effectiveness of healthcare. Recognition of the fact that patients and their families are living in difficult and unsafe circumstances, the impact of social deprivation on their health, and the genuine difficulties in following ideal professional advice about diet, healthy lifestyle and medication management could help parents to feel ‘seen’ and valued. This is particularly salient when past experience has left parents feeling vulnerable to unwarranted professional assumptions that they do not care or are incapable of caring well for their children [30, 31]. Moreover, the initiative could be beneficial for the health professionals implementing it. Many clinicians care deeply about poverty and inequality, and the opportunity to develop and administer an intervention to address something they deem important has the potential to mitigate burnout and improve well-being [32–34]. These proximal benefits might also lead to future clinical benefits but, even if they don’t, they can be understood to be positive ends in themselves.

But developing and implementing interventions like the PSI will also have significant time and resource implications, and so carries opportunity costs. During development, discussing the PSI intervention in multi-disciplinary meetings used time which clinicians could have spent on other projects or priorities. The intervention itself requires clinicians to spend time during their consultations with patients discussing their social and family circumstances, time which could otherwise be spent focussing more directly on clinical ends, or which could be used to fit in more patient appointments overall. Increased referrals have the potential to drive up costs and the overall resource burden for local charities and state-funded welfare services, even if they end up decreasing the burden on the healthcare system.

Determining the all-things-considered value of an intervention like the PSI includes determining whether its benefits outweigh its costs. This is by no means a straightforward matter. Measuring the benefits and costs may be very difficult, particularly if they are subjective goods (such as well-being and trust) or accrue to diverse parts of the healthcare system or other ‘non-health’ institutions. Moreover, even if they can be adequately measured, it remains very unclear how to compare costs and benefits of very different kinds and to weigh up their relative value.

#### The value of using a QI frame

Complex ethical trade-offs and challenges relating to appropriate measurement and comparison are unavoidable features of any intervention intended to change, and ultimately to improve, healthcare practice. Treating the PSI or another intervention as a QI initiative is one way of acknowledging and managing such trade-offs. Using a QI frame signals that the costs and benefits of a project are being considered, and to some degree systematically identified and measured. Ideally, this allows decision-makers to make judgements about whether an intervention really amounts to an improvement all things considered, and to determine whether it should be continued or extended. In practice, even with very imperfect information, a QI frame means that decision-makers can be seen to be mindful of the need to systematically monitor initiatives and to balance competing considerations. Systematic measurement and evaluation of interventions does not, of course, solve all the potential problems with comparing different kinds of costs and benefits, but it does endeavour to make such comparisons well-evidenced.

In principle, QI can accommodate a wide variety of ends: it need not only consider financial costs and benefits, but also changes in, for example, clinical outcomes, patient experience and fairness. Accounts of healthcare quality tend to be relatively broad and recognise diverse ends. The definition of what counts as good healthcare, or as an improvement, can be multi-dimensional, comprising various different, including incommensurable, goods. It can also be flexible, with different accounts of healthcare quality, or different emphases, being used or combined in different contexts [28]. Of course identifying and measuring a variety of goods and ends does not in itself generate an answer as to whether an intervention is beneficial, all things considered—assessing the value of an intervention always involves making evaluative judgements, which reflect the relative priority of different costs and benefits.

In short, QI provides a language for legitimising and justifying healthcare decisions. By requiring practitioners to explicitly identify the aims of an intervention, QI approaches can help to make the reasoning behind decisions about resource distribution and priority setting transparent. By insisting on considered development and implementation of measures, QI approaches can ensure that variables are measured consistently over time, allowing for meaningful assessment of change and the impact of interventions. For these reasons, QI has acquired high status within healthcare as a source of evidence about good practice. Designing and implementing an intervention as a QI project can thus enable healthcare professionals seeking to enact something that they reasonably believe will be beneficial for their patients or for their healthcare practice in some respect. Styling an intervention as a QI project can open up funding and resources which might otherwise be unavailable, both from within healthcare institutions and from national grant-awarding organisations. It can also increase a project’s priority status, justifying staff spending time in meetings and on administrative tasks, and changes to patient pathways or clinician roles.

#### Perspective and healthcare values

Although QI can, in principle, accommodate a diverse set of values there is further complexity in the way that different values and aims are recognised, appraised and pursued in healthcare. Different goods are valued and prioritised differently by different actors, depending on how they are positioned with respect to the healthcare system, their role and responsibilities, and their interests.

Those responsible for budgets and resource management within healthcare institutions operate within institutional and budgetary constraints and have responsibilities for values that are recognisable to and measurable by the institution, such as clinical effectiveness and financial efficiency. There are more-or-less well-defined activities that these professional actors are expected and required to carry out, and more-or-less well-defined financial resources for those activities. As such, some services and outcomes will be clearly within their remit, and so denoted ‘health-related,’ and others less so, or not at all so. It was for this reason that the clinicians developing the PSI justified the project to managers via evidence about the ability of poverty screening and informal referral interventions to reduce readmissions and burden on their services and generate improved clinical outcomes. Indications that the intervention could improve quality in this respect were seen as central to its perceived legitimacy by managers.

But distinctions between ‘health-related’ and ‘non-health-related’ outcomes are likely to be far less, if at all, salient for patients, who are not incentivised to draw boundaries between the remit and goals of different public institutions in the same way that professionals are. From the perspective of patients and families, the success of the PSI need not depend on it reducing the resource burden on the healthcare system, let alone a particular part of it, nor need it necessarily depend on its having health-specific personal benefits. While better health and fewer hospital readmissions or healthcare visits may very well be perceived as good outcomes by patients and families, they are not the only or primary things of value that patients and families stand to gain from engaging with local resources designed to help alleviate poverty and social deprivation. As mentioned above, the PSI might lead to better relationships with healthcare professionals, but also better well-being and more goods and resources in a broader sense: less stress and worry, more financial and welfare support, more household resources, stronger social networks, and reduced threat of domestic abuse. There is little reason for patients to distinguish cleanly between health-related and non-health-related benefits: they are more likely to be concerned with the ability of the system to sensitively and holistically support them in providing for themselves and their families, and respond to their needs such that services benefit them in ways which matter to them.

The paediatric clinicians who developed and administered the PSI sit between these two poles. Their professional identities are substantially shaped by the secondary healthcare institutions within which they work, and their personal aims and values are liable to be at least partly aligned with institutional ones. Although, in principle, all clinicians have some public health facing responsibilities and also need to be mindful of patient well-being and not simply biomedical outcomes [35], these broader concerns are only sometimes made institutionally explicit in population health remits or hybrid roles. Nonetheless, the legitimacy and value of the PSI for clinicians is not just grounded in the clinical and cost-effectiveness measures which concern their managers. The clinicians involved in the project recognise that many of their patients live in poverty, which causes, and has the potential to cause substantial, persistent ill effects on their health and well-being. As long as efforts focus on treating only the health effects of social deprivation, rather than the underlying or intermediating causes, their impact will be limited, given continued adverse background conditions. Healthcare professionals may even feel or appear complicit in a plastering over of deep social injustice.

For clinicians, a large part of the value of interventions like the PSI is that they provide an opportunity to use their relative knowledge and status to positively impact these underlying causal factors. The success of this does not depend on particular benefits accruing to their part of the healthcare system, or indeed to the healthcare system at all. Rather, it depends on the intervention producing some tangible benefit for their patients and their families. Moreover, clinicians have an interest in developing and maintaining open communication and a trusting relationship with their patients. Recognising the fact and burden of social deprivation and its widespread and harmful effects, and creating a space for it to be openly discussed within the context of the clinical encounter, could be instrumental in creating and sustaining such a relationship. Again, while good communication might lead to identifiable health benefits, these do not exhaust its value.

This exploration of the different values and benefits which healthcare—and different actors within it—can aim at and produce raises a number of familiar ethical tensions. It emphasises the competing goals of healthcare—including at institutional, biomedical, personal and population levels—which cannot always all be pursued or prioritised at once. Related tensions may also emerge between different ways of conceiving of the definition and value of health—whether in more technical biomedical terms or in more explicitly normative terms, factoring in the psychosocial well-being and personal aims of individual patients and communities. In addition, our account highlights that clinicians may have competing obligations—to their institutions, to patients, to their profession—which can pull in different directions.

While we recognise the importance of these tensions and they underpin our discussion, they are not our direct focus in this paper. Rather, our focus is on the way these tensions are indirectly managed as well as consciously negotiated through the use of quality discourses. More specifically in the remainder of the paper we argue that a QI framing combined with the power invested in healthcare managers as decision-makers, can lead to a limited set of institutional values being implicitly prioritised and built into healthcare decision-making. There are clear tensions facing professionals who want to help tackle social deprivation whilst based in clinical settings. Specifically, neither the pursuit of population health nor support for non-biomedical social goods are consistently incentivised by healthcare institutions in England, especially in secondary care. They may often even be disincentivised, when work is oriented around localised benefits and outcomes that are defined in biomedical and institutional terms, rather than broader social and personal goods.^3^ This leaves professionals who want to help tackle social deprivation from within clinical settings facing dilemmas. One way to avoid this unfortunate constriction of healthcare values is, we will suggest, by embracing a more explicit and expansive value pluralism in healthcare decision-making. This might involve becoming comfortable with irresolvable tensions between values, as well as recognising that not all benefits are readily measurable and that cost–benefit analysis can rarely provide a clear solution which is not also deeply value-laden.

### QI and the constraint of healthcare value

In this section, we argue that, as well as accommodating and enabling an expansive set of healthcare values, quality discourses can also constrain and disguise healthcare values in significant ways. In theory, definitions of ‘healthcare quality’ can be relatively open-ended but, in practice, quality discourses are likely to be much more circumscribed because they are shaped by specific institutional norms and priorities. QI, when used within an institutional framework for analysing the value of healthcare, will tend to prioritise certain goods and values over others, and to prioritise a particular, institutional, perspective. This makes it difficult to use a QI frame to adequately evaluate interventions, and can make it a powerful constraint on the aims and values that are pursued in healthcare. In particular, it can make it difficult to recognise and pursue wider social ends such as the relief of poverty and more intimate personal ends such as interpersonal relationships that are characterised by good communication and trust, and values like personal well-being, which are not ‘health-related’ in a restricted, institutional sense.

As noted above, quality discourses are potentially both broad and diverse. This expansiveness is valuable for purposes such as policy debate, system redesign or professional education and reform. Here there is scope not only to take seriously recognised dimensions of clinical healthcare quality that are sometimes neglected, such as person-centredness and equity, but also to utilise broader constructions of quality in public services including extra-clinical concerns related to personal and community well-being, or draw upon applications of quality discourses to public health practice [37]. However, it is inherently problematic to retain this expansiveness within QI practices, both because of the general demands of operationalisation and, more specifically, because of pressures from health service managerialism. QI is, as signalled above, largely a technical activity that depends on the operationalisation of quality concepts. In order to specify, plan, monitor and evaluate desired ‘improvements’ QI teams need to identify key indicators of progress. This technical emphasis is also typically embedded within the models and methods of QI which, for the most part, derive from industrial models of production [38]. The result is that the concern with systematisation and measurement in QI practice will always tend to close down the potential open-endedness of, and contestations of values within, quality discourses.

This is especially the case when QI sticks closely to the simplifying and unifying technicist logic of operationalised decision-making. It is highly improbable that there could be a coherent and practicable conception of healthcare quality that holds together the range of things that matter to all the differently placed actors whose interests are relevant to a project like the PSI. Not only must such a conception capture the aims and values of clinical institutions, but also those which characterise clinical relationships and underpin personal, family and community well-being. Such a conception of quality would have to weigh up a large number of objects of value which are incommensurable along a variety of different dimensions. For instance, some of them accrue to individuals, some to populations or groups of individuals, and some to institutions; some of them are objects of health related value and others are objects of social value more broadly; some of them are easily measured and others are not, or their measurement is deeply contested; some of them are only visible to and identifiable as valuable by people occupying a particular perspective. Weighing up incommensurable values to come to a single assessment of healthcare quality is difficult enough when the values are determinate, few, and constrained in scope. When the values are indeterminate, diverse and numerous, clear, accountable decision-making starts to look impossible.

We have, perhaps, painted an unduly narrow picture of QI and its potential to represent complexity. The field of QI has arguably been steadily evolving so as to narrow the gap between the elasticity of quality and the relative closure of QI models. QI has long had an interest in the use of ‘balancing measures’—additional indicators that pick up concerns beyond the key measures of success [39]. This degree of flexibility and responsiveness has now been supplemented by an interest in the ‘co-production’ of success criteria (as well as improvement processes) [40, 41]; a recognition of the importance of ‘soft intelligence’ and not just quantitative measurement in evaluation [42]; and more fundamental calls for the incorporation of pluralistic thinking within QI cultures [28, 43, 44]. All of these suggest directions for and cultures of QI that could combine technical with deliberative and participatory elements. They represent a critical, reforming current in QI, which has the potential to capture some of the ambitions of the clinical advocates of the PSI and the multiple, competing conceptions of success we have rehearsed, whilst at the same time acknowledging related trade-offs and incommensurabilities. One central element to a more pluralistic approach to QI is the idea that healthcare decision-makers should allow themselves to be more comfortable with unresolved incommensurability, that is, with evidence that does not in itself suggest a single, clear solution, where different goods and values pull in different directions. Legitimate decision-making, on such an approach, involves making explicitly normative evaluations which draw on and interpret a variety of different kinds of evidence, rather than supposing that solutions can be read directly off available data. In turn, QI practice could itself potentially evolve to reflect a greater degree of value pluralism, if it is actively shaped by communities oriented towards research or profession building—including those professional communities who helped develop the PSI. In short, this shift in a more deliberative direction would entail ‘opening up’ and self-consciously addressing the ethical contests that are inherent in the process of making quality judgements [45].

Having said this, the demands of QI within health services—especially those dominated by managerial currents, as seen in the English context—are liable to obscure or even obstruct overt engagement with value pluralism. In the real-world context within which the PSI was developed and is applied, the justification of the project via a QI methodology primarily means satisfying institutional and managerial concerns about cost efficiency and clinical effectiveness. Other concerns, such as broader social benefits or objects of personal and relational value are thereby side-lined. Although a QI methodology *could* prioritise non-managerial concerns, in practice this is unlikely to happen without a fundamental shift in the way that QI is used as a tool in healthcare. More holistic, ‘systems approaches’ to QI have been employed, with success, in the context of Indigenous healthcare provision, where community engagement and cross-sector working is imperative, and where there is a pressing need to address social determinants of health [46–48]. Such efforts have involved substantial changes in policy and healthcare infrastructure, which underpin participatory approaches to QI with genuine decision-making power with respect to healthcare delivery and evaluation.

One reason for the tendency to prioritise institutional and managerial concerns is simply that the institutionally defined priorities of those who occupy positions of power with respect to decision-making about resource allocation in healthcare settings are liable to shape how those decisions are made. QI can thus become a chiefly managerial tool, aimed at shaping and coordinating the behaviour of individuals to produce certain kinds of outcomes. In these circumstances it is used as part of efforts to take a ‘high level’ perspective, and to enact change and pursue defined ends and values at a system level. Of course, managers may also have other motivations and will usually have an interest in staff feeling effective and valued, and so will be to some extent concerned to reflect the priorities and values of clinicians in their resource-allocation decisions. But they also have strong incentives to prioritise some ends over others where these are specified in their role descriptions and personal performance indicators, partly because of the personal benefits and costs associated with these, such as salary, promotion, status, positive and negative working relationships, disciplinary action, dismissal, and so on. Of course, it is important to acknowledge that managerial concerns should not simply be dismissed as distortions of what matters: they represent values, priorities and norms in which institutions have a legitimate interest. But they are certainly not thereby the *only* or even the primary concerns that should shape institutional aims and priorities more broadly.

So while QI can act as an enabler to healthcare interventions, providing a language in which they may be justified and legitimated, the architecture and methodology of QI can also constrain them by prioritising certain kinds of values and ends, and the values and ends of particular actors and institutions, in that justification.

### Dilemmas for clinicians and value currency conversion

Clinicians who believe they have an ethical responsibility to respond to the social contexts of health and to act so as to alleviate the effects of poverty face a number of challenges. At the start of the paper we argued that clinical settings and relationships have affordances which mean clinicians might be able to make important contributions, some of which are less open to, or feasible for, others. If clinicians are motivated by population health and well-being, and perhaps even influenced by accounts of ‘quality in public health,’ they will start from the assumption that preventing the ill-health consequences of poverty is a ‘shared responsibility’ that entails targeting services to reduce inequalities and embedding this agenda system-wide so that it runs across all public policy [37]. They might reasonably think it ironic if this agenda should apply everywhere else in society but somehow not within health services themselves. And yet these larger societal ends are not typically recognised or incentivised within health service structures, so promoting them involves converting them into different value currencies. That is, it involves describing what is good about them in terms which can be better accepted and managed within existing cost–benefit and decision-making frameworks within healthcare services. This conversion is not without loss, however, and involves clinicians engaging in pragmatic compromises in order to pursue the things they consider valuable.

In order to promote the pursuit of ends such as the tackling of poverty, clinicians may have to effectively smuggle them in under different flags. In the case of the PSI, for example, this involved describing the benefits of the intervention in terms that would be accepted in managerial decision-making as within the institution’s remit, even though these did not represent clinicians’ primary motivation for developing the project, and were not perceived by clinicians to be the most valuable expected benefits. The use of a QI framing to justify and legitimate the PSI makes the project something of a Trojan horse—its true motivation is, at least in certain contexts, disguised or downplayed in order to ensure its smooth passage. Such subversiveness enables the pursuit of valued ends which are not adequately recognised or prioritised in managerial decision-making.

The same is arguably also true with respect to the more interpersonal benefits generated by interventions such as the PSI—their potential to improve communication and trust, and enrich clinical relationships. These plausibly have institutional benefits, insofar as the better clinical relationships have the potential to inform better clinical decision-making, but their value is not exhausted by these. It may be difficult or impossible for service quality radar to recognise inherent value of trust and good clinical relationships, though these are often tangible to clinicians and patients. These benefits are more difficult to measure, subjective, and less easy to observe, as they typically arise during clinical encounters and depend on the perspectives and interactions of the particular parties. These personal and interpersonal benefits are, like broader social benefits, unsuited to being counted as benefits for managerial decision-making because they are what we might call ‘metrically intangible’ at the managerial level. Broader social benefits are intangible at the managerial level because they fall outside of the scope of defined institutional practice, and personal benefits are intangible because they are difficult to identify, measure and understand from a more removed or ‘objective’ perspective.

The metrical intangibility at the managerial level of goods considered valuable by clinicians means that in order to get a project like the PSI accepted, clinicians have to navigate different value currencies, and make pragmatic adjustments and compromises. On the one hand, if they place too much emphasis on metrically intangible interpersonal and social goods, their initiatives are unlikely to gain credibility, or even a foothold, in health services, because their value will likely not be recognised by those who hold the purse strings. Re-describing the value of interventions like the PSI in managerial terms enables them to happen at all and so to produce the goods which clinicians and patients value—even though these are not necessarily the reasons why they have been prioritised. But, on the other hand, if clinicians place too much emphasis on currencies of efficiency linked to measurable health outcomes, they risk not only measuring the wrong thing, and setting themselves up to fail, but distorting the intervention to such an extent that it loses its point.

If the value of an intervention like the PSI lies in the personal and social benefits that it is liable to produce, and it does not produce significant or measurable managerial health service benefits, then a QI framing which focuses on managerial benefits is unlikely to judge the intervention successful. Converting the value of the intervention into a managerial value currency thus risks failing to showcase its actual benefits. However, even if an intervention that seeks to positively impact on poverty and social deprivation *does* generate managerially recognised benefits, describing its value in terms of its efficiency and effectiveness with respect to narrowly defined clinical and financial ends that are internal to a given health service risks undermining the point of the intervention. For if an initiative is appraised and funded on the basis of its system-level efficiencies, the social benefits it produces outside of the system will always be secondary to internal managerial goods. Such external benefits will be tolerated so long as they accompany internal benefits of managerial value, but they will not be valued in their own right. At the extreme, this might mean that the mitigation of poverty is no longer properly understood to be a de facto end of an initiative like the PSI.

This suggests that clinicians who develop projects using QI methodology which seek to promote broader social goods face some difficult dilemmas. They need to align to managerial ends to enough of an extent that their projects are authorised, but at the same time ensure they do not lose sight of the personal and social benefits of intervention. As well as the potential short-term costs of losing sight of these ends—that is, they end up not being optimised or promoted by the intervention—there are also potential long-term costs. By fully aligning to managerial ends, there is a risk that clinicians undermine the prospects of their own ends being recognised as valuable in the longer term. Failure to promote and publicise the objects of value that are not straightforwardly metrically tangible to their institutions risks further compounding their perceived irrelevance or relative unimportance.

## Conclusions

We have argued that limited constructions of clinical professional roles and ethics risk being reproduced by healthcare institutions. In particular we have highlighted the potential for quality discourses and practices to contribute to this process. The way the language and architecture of quality and QI tend to operate under managerial influence within health services can, we have argued, act as an obstacle to genuine broadening of clinical agendas. The objects of QI are constrained by institutional boundaries, making it difficult to operate with ends and goals that are not health-related in this constrained sense. This is not an inevitable problem—budgets and systems could be less siloed—but it is a problem in practice. Moreover, moving to partnership working which is supported by shared financial and institutional structures is liable to be very difficult and present other challenges [23, 49, 50].

Furthermore, the pursuit of managerial ends can fail to tap into clinicians’ motivation to pursue the objects of value that are more salient to them. The dominant language of healthcare QI is not very good at capturing the full range of what matters to individuals as good outcomes—it’s much better at capturing specific system-level effects within certain institutional settings, because it’s designed to operate at this level and in these settings. This seems like more of a fundamental problem that requires change in the methodological emphases within QI—perhaps, we suggest, via movement towards models of QI that more overtly combine deliberative with technical thinking, including via participatory approaches that bring diverse perspectives on what matters into view. This methodological reform would provide some room for extra-clinical and pluralistic conceptions of quality and could, perhaps, be used to qualify or supplement tendencies within health service managerialism that may otherwise inhibit or distort developments like the PSI. Without attention to and support for such reform, QI is likely to remain one of the mechanisms through which dominant health service norms—including the extensive professional and institutional separation of clinical and public health related remits and duties—are reproduced. This effectively pushes poverty ‘off limits,’ along with other legitimate but metrically intangible healthcare concerns.

We suggest that one way in which clinicians can encourage health systems to recognise the value of broader social and personal goods in their decision-making and resource allocation is to advocate for more deliberative and participatory models of quality improvement. Reclaiming QI as a tool to serve clinicians and patients, rather than merely managerial purposes, may involve compromising on some of its value as a technical, quantitatively-driven decision-making tool. This will require reducing the emphasis within QI on clear, uncontested answers about the benefit or value of interventions, and the possibility of ranking or directly comparing different interventions or systems. But, at the same time, it should greatly enhance the capacity of QI to respond to the complex practical and ethical tensions inherent in determining what counts as ‘good’ healthcare. As things stand there is the constant risk that such ethical tensions are either missed altogether or unwittingly ‘settled’ by institutional norms that have been insufficiently scrutinised. More open, deliberative conceptions of QI would also encourage a wider range of goods and values to be included in decision-making about healthcare system design and priority setting—a range of goods and values that more adequately reflects the multiple perspectives and interests of health service stakeholders, and avoids artificially disconnecting healthcare institutions and ‘health-related’ ends from civil society and broader social and personal goods.

## Data Availability

Not applicable.

## Abbreviations

PDSA: Plan-do-study-act
PSI: Poverty screening initiative
QI: Quality improvement
QOF: Quality outcomes framework
UK: United Kingdom

## Footnotes

1 We recognise that being relatively socioeconomically disadvantaged can impact health and healthcare use regardless of poverty status [1, 2]. However, in practice, low socioeconomic status and poverty are coexistent. In London, where the case study intervention that we discuss was developed and implemented, 39% of children live in relative poverty (in a household with income below 60% of the contemporary median) and 13% in material deprivation (unable to afford what are commonly regarded as essentials for life in the United Kingdom (UK)) [3].

2 Primary healthcare in the UK has historically included public health perspectives and sought to address social concerns more readily than secondary healthcare, so such critique might be more likely to occur in secondary than primary care settings.

3 Even institutions with an ostensible public health remit may be limited in their ability to effectively address the social determinants of health. For example, the ‘Quality Outcomes Framework’ (QOF)—a voluntary incentive programme for primary care organisations in England, which offers financial rewards for good practice—has a public health domain. However, QOF largely incentivises data collection (e.g. recording patients on disease registers), prescription of primary prevention medication (e.g. statins for patients with a new diagnosis of hypertension or type 2 diabetes), and behaviour change programmes (e.g. smoking cessation support) rather than interventions which address the underlying causes of these behaviours and health states [36].
